# Body Composition by Bioimpedance in Transgender Women, Cali, Colombia

**DOI:** 10.1155/bmri/8869056

**Published:** 2026-05-05

**Authors:** Ana Lucia Valenzuela-Gallego, Luis Miguel Becerra-Granados, Adriana Nathaly Castillo-Albarracin, Alén Fernández-González

**Affiliations:** ^1^ Department of Food and Nutrition, Pontificia Universidad Javeriana, Cali, Valle del Cauca, Colombia, javeriana.edu.co; ^2^ Faculty of Health and Rehabilitation, Institución Universitaria Escuela Nacional del Deporte, Cali, Valle del Cauca, Colombia; ^3^ Department of Social Sciences, Pontificia Universidad Javeriana, Cali, Valle del Cauca, Colombia, javeriana.edu.co

**Keywords:** body composition, gender, musculoskeletal mass, transgender women, waist circumference

## Abstract

**Background:**

Nutritional classification based on body composition in transgender populations poses methodological challenges for health professionals, particularly when sex‐based reference criteria are applied. Providing care with a gender‐sensitive approach while acknowledging underlying physiological differences remains a complex issue in clinical practice. Therefore, the study aimed to describe differences in body composition classification in transgender women according to the sex selected as reference for analysis.

**Methods:**

A descriptive, cross‐sectional, exploratory study was conducted using purposive sampling. Twenty‐five transgender women aged 23–66 years (mean age: 45.8 years), residents of Cali, Colombia, participated in 2022. Anthropometric measurements included weight, height, waist circumference, and body mass index (BMI). Body composition variables assessed by bioelectrical impedance analysis included lean mass, fat mass, skeletal muscle mass, total body water, visceral fat, bioelectrical impedance vector analysis (BIVA), and somatotype. Each participant was evaluated twice using sex‐specific reference criteria (sex assigned at birth and female sex). Statistical analyses included the Wilcoxon signed‐rank test, chi‐square test, and Fisher′s exact test, with a significance level of 5%.

**Results:**

When the same individuals were classified using male and female reference criteria, statistically significant differences were observed in the classification of skeletal muscle mass (*p* = 0.005), somatotype (*p* = 0.002), BIVA (*p* = 0.009), and waist circumference (*p* = 0.018). No significant differences were observed for BMI or other body composition components.

**Conclusions:**

The findings of this exploratory study highlight biomedical and methodological challenges in the interpretation of body composition in transgender women when sex‐based reference criteria are applied. The results underscore the need for cautious interpretation of anthropometric and bioimpedance outcomes and support the importance of further interdisciplinary research to inform the development of appropriate reference frameworks for the nutritional and clinical assessment of transgender populations.

## 1. Introduction

Internationally, gender identity has been increasingly recognized as a relevant dimension in health research and clinical practice, particularly due to its implications for access to healthcare services and health outcomes among transgender populations [1, 2]. In recent decades, conceptual frameworks have evolved from pathologizing diagnostic models toward approaches that emphasize gender identity as a component of human development and well‐being [3, 4]. Within this context, the availability of health interventions that appropriately accompany gender transition processes, facilitating alignment between sex assigned at birth and gender identity, has become increasingly relevant.

In trans women, the incongruence between the gender assigned at birth and the gender experienced manifests itself through psychological, physiological, and phenotypic transformations that emerge throughout the transition process [5]. Among these, feminizing hormone therapy (FHT) is a central component, whose use, particularly of estrogens and antiandrogens, has been associated with substantial changes in various cardiometabolic markers, such as serum levels of lipoproteins, triglycerides, blood pressure, blood glucose, and insulin, as well as with an increased risk of thromboembolic events and Type 2 diabetes [6, 7]. These changes may be exacerbated by behavioral and contextual factors, such as self‐medication and alcohol consumption [8]. From a morphofunctional perspective, it has been documented that hormone therapy induces characteristic changes in body composition from early stages, expressed as weight gain, variation in fat mass distribution in the android and gynoid regions, decreased lean mass, and changes in body circumference; these effects are modulated, among other factors, by body mass index (BMI) prior to the start of treatment [9, 10]. However, methodological and conceptual controversies persist regarding the optimal criteria for analyzing body composition in trans women and its possible convergence with the patterns observed in cisgender women, due to the endocrine and somatic complexity of the transition process and the absence of international consensus [11, 12].

In the Colombian context, the available literature on body composition in transgender women is limited and has been focused predominantly on sociocultural approaches rather than objective biomedical descriptions [13]. This lack of data hinders the clinical interpretation of anthropometric variables and the establishment of differential criteria for assessing body composition in this population. In response to this gap, the present exploratory study is aimed at characterizing anthropometric parameters using eight‐electrode bioimpedance analysis, comparing two analyses per participant: one based on sex assigned at birth (male) and another using the female sex category. This methodological approach allowed for a differential examination of the effect of the selected sex on the interpretation of body composition parameters, generating empirical evidence that strengthens current discussions on the validity and relevance of the reference values used in the clinical evaluation of trans women.

## 2. Methods

### 2.1. Participants and Design

This was a descriptive, cross‐sectional study [12]. Given the sample size and study design, the analyses were conducted within an exploratory analytical framework. Data collection was carried out in the Anthropometry Laboratory of the Pontificia Universidad Javeriana Cali between July and August 2021. Due to the limitations of research in the trans community, the population was selected using the “chain or network sampling” technique with the support of trans leaders. Sampling was purposive, with the participation of 25 trans women who met the study criterion and were of legal age.

### 2.2. Instruments

Each participant was interviewed using an adapted version of the structured interview on sociodemographic, health, and dietary factors (*Entrevista estructurada sobre aspectos sociodemográficos, de salud y alimentación*) by Hoyos‐Hernández et al. [13].

With respect to the anthropometric component, a format was adopted and used in triplicate according to the ISAK technique [14]. Variables for weight (kilogram), height (centimeter), waist circumference (centimeter), and BMI (kg/m2) were analyzed using a classification referred to by the WHO [15]. A scale was used to measure weight that was precise to 0.1 kg, with a maximum of 150 kg. Height was measured with a portable stadiometer, precise to 0.1 cm. Waist circumference was measured with a Lufkin W606PM tape measure.

SECA brand equipment was used for the bioimpedance component (mBCA 525, eight‐point segmental analysis for the right and left sides of the body), operating at 5 and 50 kHz and equipped with an internal self‐calibration system of 100 *μ*A (+20%, −50%). For each participant, the device required the entry of individual characteristics including age, weight, height, and ethnicity (categorized as “South American”), which are used by the mBCA′s validated internal prediction equations to estimate lean mass, fat mass, skeletal muscle mass, total body water, visceral fat, bioelectrical impedance vector analysis (BIVA) parameters, and somatotype. These variables constitute mandatory inputs for the SECA system and allow the software to adjust body composition estimations according to individual physiological characteristics.

Given the exploratory nature of the analysis and the absence of population‐specific reference values for transgender individuals, the derived body composition variables were analyzed and classified according to reference values validated for Latin American populations and incorporated within the device software [2, 16]. Each participant underwent bioimpedance assessment twice: first, using the sex assigned at birth (male) and subsequently using the female sex classification, as permitted by the device.

In order to avoid environmental or physiological variations among the participants, all bioimpedance measurements were performed at the same time, starting with a 10‐min rest in the supine position to stabilize intracellular fluids and reduce errors.

During the sampling process, verification was carried out by nutritionist coresearchers and assisted by a nutrition professional with ISAK certification, ensuring that anatomical points were marked and data was collected using the correct techniques.

### 2.3. Data Analysis

Prior to statistical analysis, information for each variable was entered into a spreadsheet using Microsoft Excel Version 2016. Data were subsequently analyzed using Stata Version 14 to obtain the percentage distribution of the classifications for each analyzed component and their respective 95% confidence intervals. The Wilcoxon signed‐rank test was used to compare differences between paired medians, the chi‐square test was applied for categorical data, and Fisher′s exact test was used when expected values were below 5. A significance level of 5% was established for all statistical tests.

BMI, lean mass, fat mass, skeletal muscle mass, total body water, and visceral fat were described numerically and subsequently categorized for the interpretation of the bivariate analysis, in accordance with the objective of the study. BMI categorization was based on World Health Organization reference criteria, as regulated in Colombia by Resolution 2465 of 2016, which establishes different BMI interpretations according to age range. Accordingly, participants were classified into two age groups (18–64 years and ≥ 65 years), and BMI was interpreted as underweight, normal weight, overweight, or obese. Bivariate analyses were performed based on BMI classification. For the remaining variables, classification was conducted using the age‐based reference scales incorporated in the SECA bioimpedance device.

Given the cross‐sectional and exploratory nature of the study, the analysis is aimed at comparing, within each participant, body composition classifications generated by the device under male and female settings, in accordance with the study objective. In this process, the SECA mBCA 525 device incorporated age, weight, height, and ethnicity as mandatory parameters in its validated multifrequency prediction equations, allowing body composition estimates to account for individual physiological differences. This approach is consistent with the intraindividual comparative framework adopted in the present study.

### 2.4. Ethics

The study was reviewed and approved by the Research Ethics Committee (REC) of the Faculty of Health Sciences at the Pontificia Universidad Javeriana in Cali, Colombia (Approval No. 007‐2021, June 26, 2021). The study was classified as low risk in accordance with current regulations. All procedures were conducted in accordance with the principles of the Declaration of Helsinki. All participants signed an informed consent form prior to their participation.

### 2.5. Use of Artificial Intelligence Tools

An artificial intelligence tool with a linguistic model (ChatGPT, developed by OpenAI) was used exclusively to support the linguistic editing of the manuscript during the review process. The tool did not generate scientific content, data, analysis, or interpretations. The study design, data capture, statistical analysis and interpretation, results, and conclusions are the sole responsibility of the authors.

## 3. Results

The mean age of the study population was 45.8 years (SD 10.6), ranging from 23 to 66 years. As shown in Table 1, 28% of participants belonged to a low socioeconomic group, and 84% reported earning less than twice the official minimum wage. Health insurance coverage was reported by 92% of participants.

**Table 1:** Socioeconomic and health characteristics.

Variable	Category	*n*	Percent	CI 95%
Socioeconomic group	Very low	7	28	12.1–49.4
	Low	6	24	9.4–45.1
	Middle‐low	11	44	24.4–65.1
	Middle	1	4	0.1–20.3
Monthly financial income	Not given	2	8	0.9–26.0
	Less than two minimum salaries	21	84	63.9–95.5
	Between two and five minimum salaries	2	8	0.9–26.0
Health insurance	Yes	23	92	74.0–99.0
	No	2	8	0.9–26.0
Diagnosed pathologies	Yes	12	48	27.8–68.7
	No	13	52	31.3–72.2
Type of pathology (*n* = 12)	HIV	6	50	21.1–79.0
	STD (sexually transmitted disease)	1	8.3	2.10–38.5
	CNCD (chronic non‐communicable disease)	5	41.7	15.2–72.3
Currently uses or used to use hormone therapy (HT)	Yes	22	88	68.8–97.5
	No	1	16.6	0.4–64.1
	Does not know or does not respond	2	8	0.1–26.0
Who prescribed the HT (*n* = 22)	Self‐medicating	19	86	65.1–97.1
	Doctor/physician	3	13.6	1.2–51.9

Regarding self‐reported health status, 48% indicated having at least one condition previously diagnosed by a health professional, with human immunodeficiency virus (HIV) accounting for 50% of the reported conditions. FHT was reported by 88% of participants, of whom 86% indicated self‐medication or support from another transgender woman.

With respect to anthropometric measures, the median values for weight, BMI, and waist circumference were 72 kg (IQR 64.1–79.0), 24.8 kg/m^2^ (IQR 22.5–27.4), and 82 cm (IQR 74–90), respectively. Mean height was 169 cm (SD 7.2).

Tables 2 and 3 present the results of the bioimpedance analysis using sex‐differentiated classification criteria for each assessed body composition component. Within this exploratory sample, no statistically significant differences were observed in the percentage distribution of lean mass, fat mass, skeletal muscle mass, or total body water when classified according to sex (Table 2).

**Table 2:** Body components according to sex and quantitative data.

Component	Sex		*p* value^a^
	M	F	
% of lean mass (median/RI)	75.5% (RI 69.3–84.7)	72.3% (RI 65.8–79.2)	0.2563
% fat mass (median/RI)	21.8% (RI 15.3–30.7)	27.7% (RI 20.8–34.2	0.1713
% skeletal muscle mass (mean/DS) (*n* = 24)	37.7% (DS 17.8–58.3)	35.3% (DS 20–50.1)	0.2160
% of total body water (median/RI) (*n* = 24)	57.1% (RI 51.1–61.1)	53.9% (RI 49.1–58.3)	0.2836

^a^Wilcoxon test: *p* value.

**Table 3:** Body components according to sex and categorical data.

Component	Category	M		F		*p* value
		% (CI 95%)		% (CI 95%)		
BMI	Thinness	1	4 (0.1–20.3)	1	4 (0.1–20.3)	NA
	Normal	14	56 (34.9–75.6)	14	56 (34.9–75.6)	
	Overweight	7	28 (12.1–49.4)	7	28 (12.1–49.4)	
	Obesity	3	12 (2.5–31.1)	3	12 (2.5–31.1)	
Lean mass index (kg/m^2^)	Normal	21	84 (63.9–95.5)	23	92 (73.9–99.0)	0.334^a^
	Low	4	16 (4.0–36.0)	2	8 (0.9–26.0)	
Fat mass index (kg/m^2^) (*n* = 24)	Normal	10	41.7 (22.1–63.3)	9	37.5 (18.8–59.4)	0.083^b^
	Low	2	8.3 (0.10–27.0)	8	33.3 (15.6–55.3)	
	Elevated	12	50 (29.1–70.9)	7	29.2 (12.6–51.1)	
Skeletal muscle mass (*n* = 24)	Normal	16	66.7 (44.7–84.4)	4	16.7 (4.7–37.4)	**0.002** ^**b**^
	1 low	3	12.5 (2.7–32.4)	10	41.7 (22.1–63.4)	
	2 elevated	5	20.8 (7.3–42.2)	10	41.7 (22.1–63.4)	
Somatotype (*n* = 23)	Ectomorphic	11	47.8 (26.8–69.4)	2	8.7 (1.0–28.3)	**0.002** ^**a**^
	Endomorphic	6	26.1 (10.2–48.4)	3	13 (2.8–33.6)	
	Mesomorphic	6	26.1 (10.2–48.4)	18	78.3 (56.3–92.5)	
Total body water (%) (*n* = 24)	Low	24	100	24	100	NA
BIVA (bioelectrical impedance vector analysis) (*n* = 24)	Increased cell mass	1	4.2 (0.1–21.1)	0	0	**0.009** ^**a**^
	Increased muscle mass	1	4.2 (0.1–21.1)	0	0	
	Increase in the proportion of water	17	70.8 (48.9–87.4)	24	100	
	Decrease in cell mass	2	8.3 (1.0–27.0)	0	0	
	Decrease in the proportion of water	3	12.5 (2.6–32.4)	0	0	
Visceral fat (VAT I) (*n* = 24)	Normal	16	66.7 (44.7–84.4)	12	50 (29.1–70.9)	0.242^b^
	Elevated and high	8	33.3 (15.6–55.3)	12	50 (29.1–70.9)	
Waist circumference	Normal	20	80 (59.3–93.2)	12	48 (27.8–68.7)	**0.018** ^**b**^
	Elevated	5	20 (6.8–40.7)	13	52 (31.3–72.2)	

*Note:*Values in bold correspond to statistically significant differences (*p* < 0.05).

^a^ *p* value from chi‐square test.

^b^Fisher′s exact test.

In contrast, analysis of categorical body composition outcomes derived from bioimpedance revealed statistically significant differences in skeletal muscle mass classification (*p* = 0.002), somatotype (*p* = 0.002), BIVA (*p* = 0.009), and waist circumference (*p* = 0.018) when the same individual was classified using male versus female reference criteria (Table 3). Given the limited sample size and the lack of adjustment for age variability and self‐reported hormone use, these findings should be interpreted as preliminary and exploratory.

With regard to the categorical body composition data obtained from bioimpedanciometry, statistically significant differences were observed in skeletal muscle mass (*p* = 0.002), somatotype (*p* = 0.002), BIVA (*p* = 0.009), and waist circumference (*p* = 0.018) within the same individual when classified as male (M) or female (F) (see Table 3).

## 4. Discussion

In accordance with the exploratory scope of the study, the assessment of body composition using bioimpedance analysis, with specific cutoff points for each sex, revealed statistically significant differences in the classification of skeletal muscle mass, somatotype, waist circumference, and BIVA when participants were analyzed according to male or female categories. In contrast, no differences were observed in the classification of BMI, given that the World Health Organization criteria were applied for this variable, which does not differentiate by sex [15, 17]. Taken together, these findings highlight the sensitivity of body composition interpretation in relation to the selected gender reference framework in transgender women and should be interpreted with caution due to the exploratory design and small sample size.

In relation to lean mass and skeletal muscle mass, when using male reference values, a higher proportion of participants were classified as below normal or within average ranges, compared to the female classification. These findings may reflect changes associated with FHT, particularly the reduction in testosterone levels and its effect on skeletal muscle [4, 8, 18–22]. However, the lack of detailed information on the type, dose, route, and duration of hormone treatment, as well as the self‐reported nature of this information and the wide age range of the participants, limits the possibility of establishing causal relationships and reinforces the preliminary nature of these results. The assessment of fat mass did not show statistically significant differences according to the reference sex, although a marginal trend (*p* < 0.1) and a higher proportion of low fat mass index classifications were identified when using female reference values. These variations could be influenced by factors such as food intake, physical activity, and FHT [8, 23, 24]. Although these variables were not strictly controlled in the analysis, they were investigated in the study and have been consistently associated with changes in body composition in the literature. In this context, the findings provide preliminary evidence on the influence of hormone therapy on the body composition of trans women and reinforce the existing gap in the literature, where methodological limitations persist, including the lack of specification of the reference values used for anthropometric classification.

The analysis of somatotype and BIVA showed statistically significant differences when applying gender‐specific reference criteria. In the case of somatotype, ectomorphic profiles were more frequent under the male classification and mesomorphic profiles under the female classification, while BIVA showed relevant changes in the classification of components such as cell mass, muscle mass, and body water. Given that both methods depend on ranges of normality defined by sex and in the absence of reference values specifically validated for the transgender population, these results should be interpreted with particular caution.

Body water distribution also showed marked variability according to reference sex. Although water balance is influenced by hormonal factors, including exposure to estrogen, as well as by protein‐mediated fluid regulation mechanisms [25, 26], the present study does not allow us to establish clinical implications associated with these differences or to discriminate between physiological redistributions and pathological alterations.

Finally, waist circumference and visceral fat index, both indicators related to cardiovascular risk [27, 28], showed statistically significant differences when specific cutoff points were applied by sex. When using female reference values, a higher proportion of participants were classified as having increased cardiovascular risk, reflecting the lower thresholds defined by international guidelines [29]. However, these results should be interpreted with caution, as the study was not designed to evaluate clinical outcomes or actual cardiovascular risk.

Overall, the findings of this study highlight the complexity of assessing body composition in transgender women when gender‐specific reference standards are applied. The main limitations include the small sample size, nonprobability sampling, wide age range, use of self‐reported hormone therapy, and absence of specific reference values for the transgender population, which limits the generalizability of the results. In this context, the findings should be considered preliminary and hypothesis‐generating, highlighting the need for future studies with larger samples, longitudinal designs, and standardized characterization of hormone therapy to advance toward more appropriate reference frameworks for assessing body composition in transgender women.

## 5. Conclusion

The assessment of body composition in transgender women, based on the differentiation between sex and gender, poses significant biomedical and methodological challenges for anthropometric classification using bioimpedance. The findings of this exploratory study show that the selection of the reference sex influences the classification of variables such as skeletal muscle mass, somatotype, visceral fat, and BIVA, while other components do not show significant variations, regardless of the reference criterion used. These results highlight the complexity of interpreting body composition in this population and the limitations of directly applying reference frameworks designed for cisgender populations.

Given the exploratory design of the study and its methodological limitations, the findings should be interpreted with caution and are not generalizable. However, they provide preliminary evidence that contributes to the discussion on the relevance of the criteria used for assessing nutritional status and body composition in trans women, especially in the context of hormonal transition processes and associated physiological variability.

Furthermore, considering the clinical and social particularities that characterize the transgender population, as well as the historical barriers to access to health services and standardized clinical assessments, the results of this study highlight the importance of strengthening interdisciplinary research and moving toward nutritional and clinical assessment approaches that are more sensitive to these realities. In this regard, future studies with longitudinal designs, larger samples, and standardized criteria are needed to contribute to more accurate, individualized, and evidence‐based healthcare for this population.

## Funding

This study was supported by the Pontificia Universidad Javeriana, Cali, Colombia (Investigar PUJ1528).

## Conflicts of Interest

The authors declare no conflicts of interest.

## Data Availability

The data supporting the findings of this study are not publicly available due to ethical considerations related to the sensitive nature of the study population and the potential risk of reidentification, given the sample size. Additionally, data availability was not contemplated in the protocol approved by the ethics committee, which establishes strict commitments to confidentiality, privacy, and the protection of participants, in accordance with the provisions of the informed consent form. Regarding this, even access to anonymized data is restricted. However, additional methodological information may be provided by the corresponding author upon reasonable request.
